# Notes and News

**Published:** 1891-07-18

**Authors:** 


### Notes and News.

Collected and Communicated.

It has been decided that the collectors of the Hospital Saturday Fund to-day shall wear a small silk-woven badge, bearing the registered cross of this association, and having the words "Hospital Saturday Fund Streets Collection, July 18, 1891," thereon.

Our frivolous contributor writes that he cannot find more than an iota of difference between hay-fever and influenza. One, he says, often results from "new-mown hay," and the other often results in "pneumon(i)a"! We should like to suggest that Berrywood is a very comfortable asylum.

A distinguished statistician, jumping out of his brougham a few nights ago, jammed his head against the top of the door, and the spring of his opera hat penetrated the scalp. The wound bled freely, but was not dangerous, had not some well-meant friends dressed it with pepper. Their notion was to stop the bleeding with flour, but unfortunately they seized the pepper-pot instead of the flour-dredger; the result was not only great pain to our new C.B., but a certain amount of danger from inflammation. This proves that wider knowledge of "First Aid" is most advisable in all circles.

The dissatisfaction felt towards street collections in connection with charities is on the increase. In towns, and especially in the metropolis, such a system of collections is open to many objections. It places temptation in the hands of a class of persons not calculated to be able to resist it, even should the public be satisfied as to the *bona fide* nature of their appeals. The inadequate response met with may be gathered from the fact that the outlay on these out-door collections is stated by the *Pictorial World* to amount to fifty per cent. of the proceeds.

We cannot congratulate the General Practitioners' Alliance upon the statements made, apparently without contradiction, of the abuses to which hospitals are subjected. To bring up again the case of the "well-known lady" who drives in her carriage to a bye-street near the hospital, in order that she may enter and receive free medical relief, is a myth as old as the institutions of this country. How many times the statement has been made and refuted on the evidence it would be difficult to say. It says little for the wit of the gentlemen present that someone did not get up and remind the audience of the antiquity of this assertion at any rate. We should like to have a few more particulars of the man who died worth £50,000, and was continually treated in one of the voluntary hospitals. May we ask for the gentleman's name, the date of his death, and the institution referred to? No one sympathises more than we do with the hard life which many general practitioners have to lead, but they will not be helped by statements of this character unless chapter and verse are given, and the speakers are prepared to submit to cross-examination. Any abuses that can be proved can be remedied. Mere assertion only injures the voluntary charities without benefitting the general practitioners, and so should be discountenanced by all intelligent people.

The report for 1890 of the Metropolitan Provident Medical Association is just issued. The Association has now sixteen branches open, of which twelve are dispensaries and four medical clubs. There are 25,000 members representing 8,300 families, being an average of three members to each family. Of the sixteen provident dispensaries open, nine are now self-supporting, and two others will probably prove to be so during the year. A committee has been formed to consider the best methods of promoting the increase of provident dispensaries throughout London. Some of the hospitals, including the Middlesex, the Westminster, and the Royal Free, have elected members on the committee of the Association, and have allowed its bills to be hung in the out-patients' departments. The balance-sheet shows a balance in favour of the Association of £411.

It is not a pleasing fact that receiving and giving can be acted upon in reverse ratio. It would seem that the advance in wages has not had a beneficial effect upon the generosity of the Wakefield working population. In the last annual report presented at the annual meeting of the Clayton Hospital we find that the contributions from workshops and collieries has undergone a diminution in the past year. The workshops contributed £418 against £450, and the collieries £300 as against £303 during the former period. It is fair to state, however, that the Hospital Saturday collections are steadily increasing. In all other respects the report is a favourable one. A proposal to erect a memorial tablet in the entrance hall of the hospital to Mr. Binks, the late secretary of the hospital, was unanimously adopted.

The town of Nottingham is fortunate in possessing so generous a friend as Colonel Seely (late High Sheriff of the county), whose generous initiative has met with a liberal response. The want of convalescent homes situated outside the town, and of a convalescent hospital within the town, have been long felt. Through the generosity of Colonel Seely, Nottingham is now in possession of three such institutions. Towards the establishment of the homes he has contributed the sum of £10,000, and has promised £200 annually for five years in aid of the convalescent hospital. This last institution was opened by Mrs. Seely, and visitors present at the ceremony expressed themselves much pleased with the arrangements. All three establishments are connected with the Nottingham General Hospital, the authorities of which will undertake the management and direction.

Hospital Saturday in Birmingham has been established nineteen years. The yield in the first year, 1873, amounted to £4,700. Then it dropped gradually until in 1878, it stood at £2,900, the net amount available for division. From that period, the produce of the collection began to rise, each successive year, until in 1890 the fund to be divided amongst the medical charities amounted to £9,400. The managers, however, had set their hearts upon £10,000. This year their desire has been fulfilled and their labour rewarded by a collection which yielded the magnificent total of £10,867 19s. 3d.; after payment of expenses, and keeping a small reserve there remained £10,300 for division amongst the charities. The *Birmingham Post* justly says "Looking to the total amount yielded by this collection from its beginning, we find from this year's report that from 1873 to 1891 the sum of over £107,000 has been contributed by the working classes of Birmingham as a free gift to the hospitals of the city. Compared with similar movements in other places this record confers upon Birmingham a distinction which is unique.

At a meeting of the General Committee of the Birmingham Hospital Saturday Fund a proposal emanating from Mr. Smedley to erect a convalescent home in connection with the fund, was received, considered, and referred to a Committee. The details of the scheme, of course, have yet to be carefully worked out, but it is well conceived and should prove a great success.

